# Vascular risk factors affect different brain regions in people with Alzheimer’s disease

**DOI:** 10.1192/j.eurpsy.2022.851

**Published:** 2022-09-01

**Authors:** S. Tripathi, A. Murray, C. Wischik, B. Schelter

**Affiliations:** 1 King George’s Medical University, Department Of Geriatric Mental Health, Lucknow, India; 2 University of Aberdeen, Aberdeen Biomedical Imaging Centre, Aberdeen, United Kingdom; 3 University of Aberdeen, School Of Medicine, Medical Sciences And Nutrition, Aberdeen, United Kingdom; 4 University of Aberdeen, Institute Of Complex System And Mathematical Biology, Aberdeen, United Kingdom

**Keywords:** brain imaging, Vascular risk factors, [18F] fluorodeoxyglucose- positron emission tomography (FDG-PET), Alzheimer’s disease

## Abstract

**Introduction:**

Vascular risk factors including hypertension, diabetes and dyslipidaemia promote diverse pathological mechanisms in the brain leading to cerebral hypoperfusion and ultimately cognitive decline in people. Medial temporal, medial frontal and anterior cingulate atrophy has been closely associated with diabetes and medial temporal lobe atrophy is associated with hypertension in people with Alzheimer’s disease (AD).

**Objectives:**

To assess if hypertension, diabetes and dyslipidaemia have differential effects on different brain locations using brain imaging in people with AD.

**Methods:**

The current study is based on [^18^F] fluorodeoxyglucose- positron emission tomography (FDG-PET) data of 970 participants from two large Phase III multi-centre clinical trials of a novel tau aggregation inhibitor drug Leuco-Methylthioninium (LMTX)meeting research criteria for mild to moderate AD. Vascular risk factor data including hypertension, diabetes and dyslipidaemia were collected and quantification of FDG PET hypo-metabolism was done by calculating Standardized Uptake Value Ratio(SUVR).

**Results:**

Hypertension, diabetes and dyslipidaemia were found to have differential effects on brain locations in people with AD. When people with hypertension, diabetes and dyslipidaemia were compared to those without, mean SUVR was increased significantly in both left and right parietal and occipital lobes and decreased in left and right anterior cingulate gyri in hypertensives. SUVR was significantly higher in both left and right temporal lobes in diabetics andlower in both left and right anterior cingulate gyri in people with dyslipidaemia.

**Conclusions:**

Vascular risk factors including hypertension, diabetes and dyslipidaemia have differential effects on different brain regions, measured using SUVR analysis of FDG-PET.

**Disclosure:**

The FDG-PET data was taken from participants of two large phase III clinical trials sponsored by TauRx Therapeutics (Singapore). TauRx Therapeutics has contributed towards my studentship during my PhD but the data related to drug used in the clinical tria

